# Daily Rhythms in Mobile Telephone Communication

**DOI:** 10.1371/journal.pone.0138098

**Published:** 2015-09-21

**Authors:** Talayeh Aledavood, Eduardo López, Sam G. B. Roberts, Felix Reed-Tsochas, Esteban Moro, Robin I. M. Dunbar, Jari Saramäki

**Affiliations:** 1 Department of Computer Science, Aalto University School of Science, Espoo, Finland; 2 CABDyN Complexity Center, Saïd Business School, University of Oxford, Oxford, United Kingdom; 3 Department of Psychology, University of Chester, Chester, United Kingdom; 4 Department of Sociology, University of Oxford, Oxford, United Kingdom; 5 Departamento de Matemáticas & GISC, Universidad Carlos III de Madrid, Leganós, Spain; 6 Department of Experimental Psychology, University of Oxford, Oxford, United Kingdom; University of Namur, BELGIUM

## Abstract

Circadian rhythms are known to be important drivers of human activity and the recent availability of electronic records of human behaviour has provided fine-grained data of temporal patterns of activity on a large scale. Further, questionnaire studies have identified important individual differences in circadian rhythms, with people broadly categorised into morning-like or evening-like individuals. However, little is known about the social aspects of these circadian rhythms, or how they vary across individuals. In this study we use a unique 18-month dataset that combines mobile phone calls and questionnaire data to examine individual differences in the daily rhythms of mobile phone activity. We demonstrate clear individual differences in daily patterns of phone calls, and show that these individual differences are persistent despite a high degree of turnover in the individuals’ social networks. Further, women’s calls were longer than men’s calls, especially during the evening and at night, and these calls were typically focused on a small number of emotionally intense relationships. These results demonstrate that individual differences in circadian rhythms are not just related to broad patterns of morningness and eveningness, but have a strong social component, in directing phone calls to specific individuals at specific times of day.

## Introduction

Human activity follows a circadian rhythm that is reflected at the psychological, physiological and biochemical levels [1–3]. This rhythm is mainly driven by endogenous cellular mechanisms, but it may be modulated by exogenous factors. Circadian rhythms are in general synchronized to the day-night cycle. However, within this cycle, there are differences between individuals, and individuals’ levels of alertness vary following different trajectories throughout the day. Notably, there are morning and evening types, those who tend to wake up early and those who prefer to sleep late. This may result from intrinsic differences in the circadian pacemaker circuit, possibly of genetic origin. Morningness and eveningness of individuals have also been associated with gender and personality traits [4–6]; typically, these studies have been conducted with the traditional methods of surveys and questionnaires. In studies of statistics aggregated over large numbers of individuals, circadian patterns and daily cycles have been observed in a wide range of phenomena: in human suicidal acts, in times of exhibiting unethical behaviour, in times of sexual activity, and in times of heart attacks [7–10].

Aggregate-level daily rhythms are also known to appear in electronic records of human activity, from mobility [11] to Wikipedia and OpenStreetMap edits [12, 13], activity on Twitter [14], and the number of mobile phone calls per hour [15, 16]. In addition to the day-night cycle, these patterns are modulated by a number of endogenous factors such as the daily work schedule, commuting patterns [16], the activity patterns of one’s social circles, and even one’s life circumstances such as employment or unemployment [17]. In general, in such studies of daily rhythms inferred from electronic records, the focus has typically remained at the aggregate level.

In this paper, we use a unique longitudinal dataset [18, 19] that combines questionnaire data with mobile phone records, allowing us to take an individual-centric point of view instead of focusing on aggregates. In particular, since the activity levels of individuals are known to display individual differences, in particular morningness and eveningness, we want to see whether there are also clear individual differences in daily call frequency patterns. Further, we want to address the persistence of these differences. First, this is to guarantee that individual differences are real and not only because of random fluctuations giving rise to diversity. Second, we also use the concept of persistence to address the issue of intrinsic versus exogenous drivers. Our data set has been collected during a period of high network turnover for the individuals, and thus if daily patterns are seen to persist, they cannot be purely driven by one’s social network. In addition, we want to uncover details of the observed patterns and to look for “patterns within patterns”: are certain times of day reserved for calling certain alters, or are calls typically placed to random individuals? Do the properties of callers and callees, such as gender, explain some features of the observed patterns?

To this end, we employ a data set that comprises the exact times and recipients of all outgoing mobile phone calls of 24 individuals (“egos” in the following) for 18 months. This data set consists of 74,124 phone calls altogether. This set was originally collected for the purpose of studying social network turnover over time [18]: during the study, the participants finish high school and go to work or university (often in another city), which gives rise to major turnover in their personal networks. At the beginning of the study, the students are still at high school. Thereafter, they begin their first university year or go to work at around month 6 of the study (after the summer holiday period). This gives rise to turnover, and in general, should result in rather different exogenous factors affecting the daily patterns from the beginning to the end of the study. This is also reflected in an increase in the total number of calls made by the participants from around month 6 (see Fig 1 in [19]). The mobile phone call data set is accompanied by 3 surveys on the contacts who were called (“alters” in the following), including their gender and information on kinship.

Together, these data allow us to study the daily rhythms of calls in terms of numbers and in terms of recipients (who is called and when). We find that in terms of call frequency at each hour of day, each individual has their distinct, persistent pattern. These daily patterns persist for individuals despite a high degree of network turnover, and thus appear to be characteristics of individual egos, rather than dependent on the identify of specific alters. Within these patterns, there are clear variations in the entropy of called alters, indicating that certain times of day (evening and night, typically) are reserved for calling specific alters, whereas at other times the recipients of calls are more diverse. For female egos, there is an additional increase in the average duration of calls towards the night—these long calls are typically made to friends instead of family members.

## Results

### Persistence of individual daily call patterns

We begin by computing the daily call patterns for all 24 egos. The data time span is divided to three consecutive 6-month intervals *I*_1_, *I*_2_, and *I*_3_. For each ego and each 6-month interval, we compute the average fraction of calls placed at each hour of the day. Considering 6-month intervals separately allows investigation of the persistence of any observed differences: were specific features of individual patterns due to random fluctuations alone, they would not persist over all intervals.

**Fig 1:**
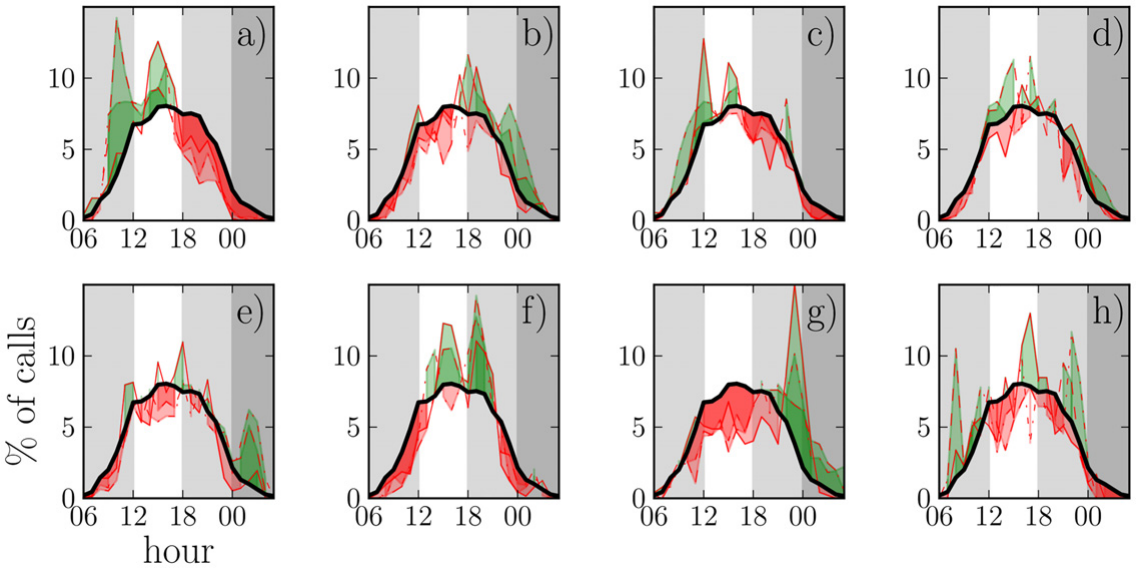
The daily call patterns of 8 individuals (a-h). The red lines denote the average fraction of calls placed at the corresponding hour for each of the three intervals *I*_1_ (solid line), *I*_2_ (dashed line), and *I*_3_ (dash-dotted line). The black line is the average call pattern of all 24 individuals over all intervals. Areas shaded green show where an individual’s fraction of calls exceeds the average, while areas shaded red show where it falls below the average.

The resulting daily call patterns for 8 representative egos (4 male, 4 female) for all intervals are displayed in Fig 1. Two features clearly stand out: First, while the call patterns of all egos follow the day-night cycle and calls at night are infrequent, there are significant differences between individuals. As an example, the ego whose pattern is displayed in panel a) makes more calls in the morning than others, whereas for the ego of panel g) there are frequent calls at late hours. Second, it appears that each individual’s specific patterns are rather similar in all 6-month intervals. Both observations hold for all 24 egos. This persistence is noteworthy, since it is known that at the same time, the social networks of these individuals undergo major turnover [19]. Because of this, the observed persistence points towards intrinsic driving forces behind the daily patterns, as these do not strongly depend on an ego’s personal network composition.

**Fig 2:**
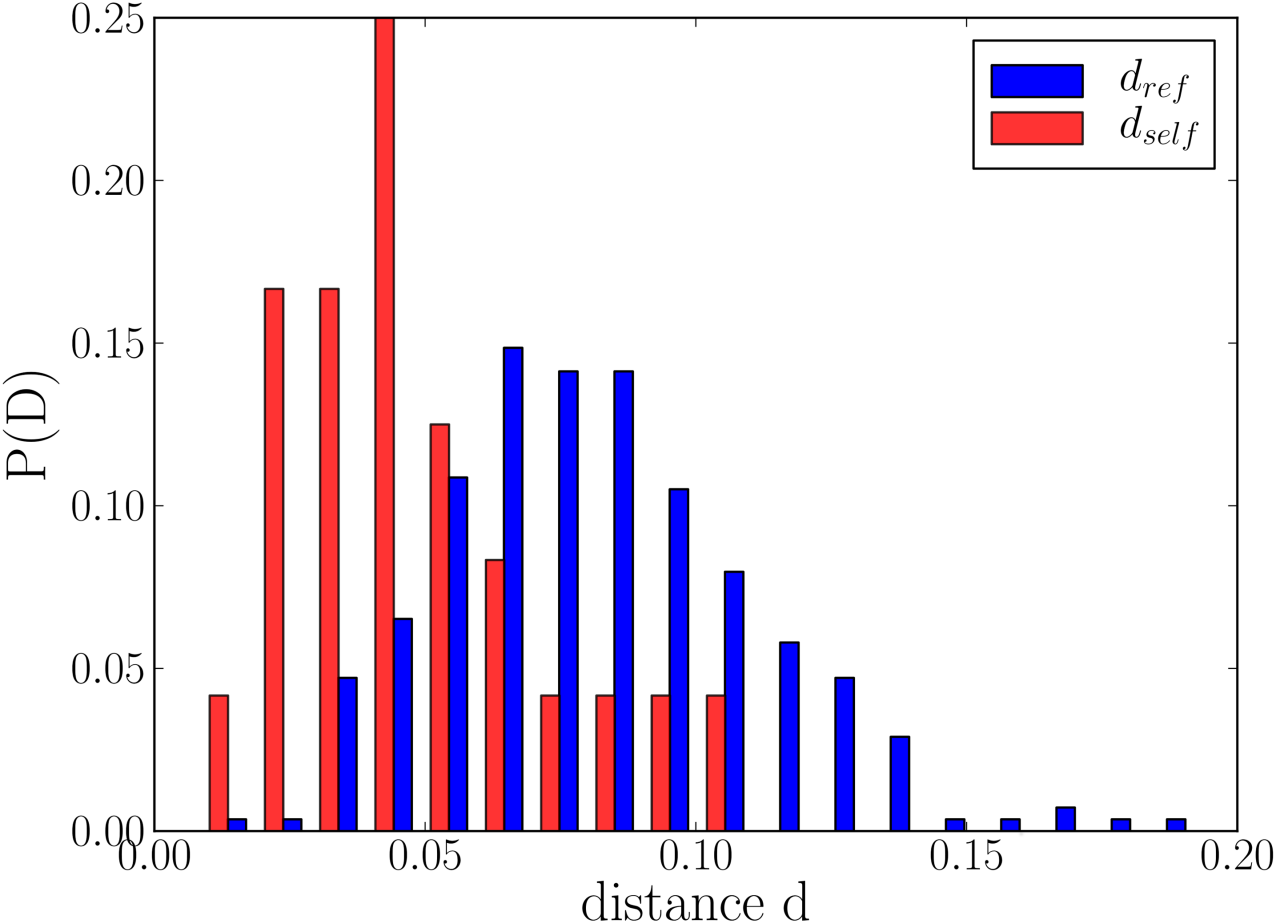
Histogram of *d*_self_ and *d*_ref_ calculated for each ego. This plot shows the results for all egos and all time intervals.

The persistence of individual daily patterns is confirmed with a more detailed analysis. Here we use the approach of Ref. [19] to show that the daily call patterns of an individual in different time intervals are more similar than the patterns of different egos within one time interval. We use the Jensen-Shannon divergence (JSD) (see Materials and Methods for details) to measure the difference between daily call patterns. For each ego, we calculate two different distances: self (*d*_self_) and reference (*d*_ref_). The self-distance *d*_self_ for an individual *i* is the average JSD between the call patterns in (*I*_1_, *I*_2_) and (*I*_2_, *I*_3_): $d_{i,\text{self}}=\frac{1}{2}\;(d_{12}^{i,\text{self}}+d_{23}^{i,\text{self}})$. The reference distance measures the divergence of patterns of different egos in one time interval. For each time interval we calculate JSD between daily patterns of egos *i* and *j*: $d_{\text{ref}}^{ij}=\frac{1}{3}\;(d_{11}^{ij}+d_{22}^{ij}+d_{33}^{ij})$. As seen in Fig 2, *d*_self_ takes on average lower values than *d*_ref_, meaning that there is more similarity between an ego’s consecutive daily patterns than between the patterns of different egos in one interval. The motivation behind this approach is as follows: while an ego’s consecutive patterns retain their overall shapes well (Fig 1), they are not exactly similar and there are a lot of fluctuations. Therefore, the aim is to look for persistence by checking whether consecutive patterns of an ego show a high level of similarity; however, to assess whether similarity is high or low a scale is required. The natural scale to use is that given by the similarities between patterns of all egos; if one ego’s pattern remains more similar to itself than it is to others, we call it persistent. On average, for each ego, 87% ± 12% of reference distances are higher than self-distances. Comparing average values of distances over all egos we get 〈*d*_ref_〉 = 0.083 ± 0.28 while 〈*d*_self_〉 = 0.05 ± 0.22 (〈*d*_self_〉 < 〈*d*_ref_〉 with *t* = 6.98 and *p* ≪ 10^−6^, two-sample unequal variance t-test). To validate these results with another method, we have used the ℓ^2^ norm (see Methods), and the results qualitatively agree with JSD: 〈*d*_self_〉 = 0.11 ± 0.02 and 〈*d*_ref_〉 = 0.14 ± 0.03 with *t* = 6.11, *p* < 10^−5^. We also used the Kolmogorov-Smirnov test on each ego’s successive patterns, and found that the null hypothesis of patterns being similar cannot be rejected in 44 out of 48 cases with confidence level *p* = 0.01.

As an alternative to the above, we have verified the persistence of patterns with coarse-grained daily patterns and a distance measure whose values are easier to interpret directly. To this end, we begin by coarse-graining the patterns and compute the fraction of calls placed in 6-hour bins (night: 0AM–6AM, morning: 6AM–12AM, afternoon: 12AM–6PM, evening: 6PM–12PM) for each ego and each 6-month interval. Then, using all egos, we generate a common reference pattern by computing the *median* fraction of calls in each 6-hour bin, either a) for the whole 18-month interval or b) separately for each 6-month interval. We then take each ego’s pattern in each interval, and for each 6-hour bin, check whether the fraction of calls is above the median (‘+’) or below the median (‘−’). This yields a string of four characters for each ego and interval, e.g. ‘++−−’ denotes that for the focal ego and interval, the fraction of calls is above median at night and in the morning, but below median in the afternoon and evening. Then, we compute the Hamming distances between each ego’s strings in different intervals, where a distance of 0 means that the strings are identical and a distance of 4 means that all characters differ. For both a) and b), *i.e*. 18-month or interval-specific median references, the outcome is that the average Hamming distance is *d*_H_ = 1.1 characters, which is low (note that the fraction of calls in a bin is categorized as above median (‘+’) even if it exceeds the median by a vanishingly small amount, so we do not expect zero distances). Were the patterns changing randomly independently of one another, this average distance would be *d*_H,ref_ = 2.0; taking the distributions of Hamming distances for the observed patterns and the multinomial distribution centered around 2.0, the difference in means is significant with *p* < 10^−5^.

### Alter-specificity in call patterns

We next turn to the question of where the individual daily patterns come from, and study the extent of a social component -that is, alter-specificity- in the call patterns. One can conceive of two extreme cases: 1) The patterns are entirely endogenous and the rate of call activity at each hour of the day is intrinsic to the ego. In this case, the called alters are picked at random (however, with a weight proportional to the time-averaged fraction of calls to each alter). 2) The patterns are alter-specific, that is, calls to certain alters are placed at certain hours, and the daily pattern is a superposition of the alter-specific patterns.

To assess the extent of alter-specificity, we again divide the day into 6-hour time spans (night: 0AM–6AM, morning: 6AM–12AM, afternoon: 12AM–6PM, evening: 6PM–12PM), and for each alter and each time span, compute the relative call entropies *H*_rel_. First, call entropies *H*_orig_ are calculated from the original data for each 6-hour span. To get the relative entropies *H*_rel_, these values are then divided by average entropies 〈*H*_ref_〉 calculated for a reference model where the times of calls to all alters are shuffled on a weekly basis for each ego (see Methods). If for a given 6-hour span *H*_rel_ < 1, calls to certain alters are emphasized within that time span, whereas if *H*_rel_ ≈ 1, there is no alter-specificity. Note that the relative entropies can be *H*_rel_ > 1. This can happen because of the following: suppose that for some hour, calls are placed evenly on a number of alters in the original data, resulting in high entropy. At the same time, some other alter (‘A’) receives very many calls outside this hour. Then, when the data are shuffled, this alter ‘A’ replaces many of the alters of the high-entropy hour, *i.e*. now several of the calls during that hour are directed at ‘A’. Because of this, the average entropy of this hour is then lower in the shuffled data, and subsequently, *H*_rel_ > 1.

**Fig 3:**
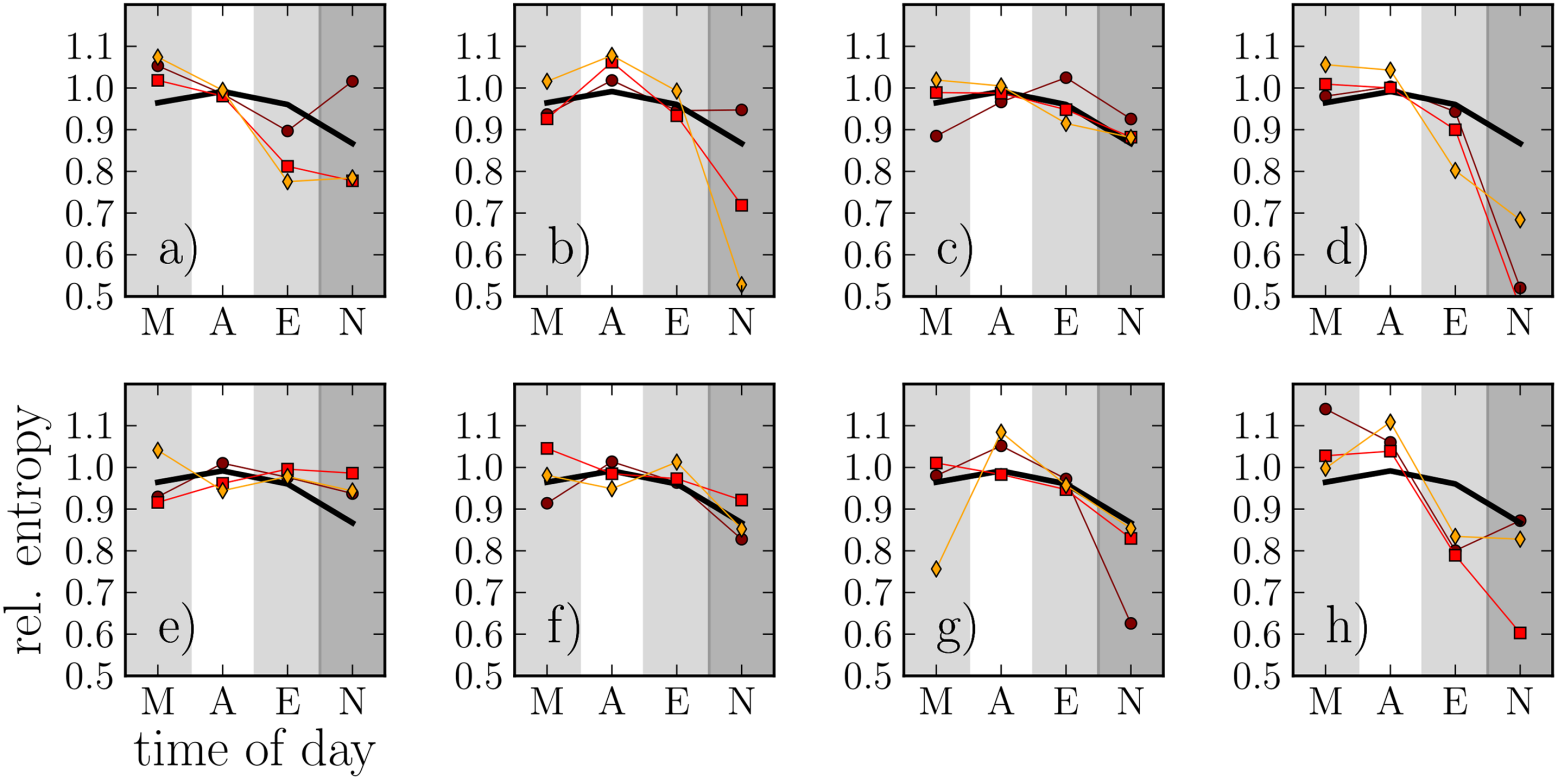
The relative entropies for the same 8 individuals as in Fig 1, calculated for 6-hour intervals (M: morning 6AM-12AM, A: afternoon 12AM-6PM, E: evening 6PM-0AM, N: night 0AM-6AM). (ο): interval *I*_1_, (◻): interval *I*_2_, (◊): interval *I*_3_. The black line indicates the average relative entropy for all 24 individuals over all three intervals.

The relative entropies *H*_rel_ for the same 8 individuals as in Fig 1 are displayed in Fig 3, together with averaged relative entropy for all 24 individuals over all three intervals. The average relative entropy is at its highest in the afternoon, with 〈*H*_rel_〉 ≈ 1, indicating large diversity of called alters. 〈*H*_rel_〉 has its lowest point at night, when the number of calls is also low (see Fig 1). This indicates that the few calls made at night are typically directed to specific alters. As with the call frequency patterns, Fig 1 clearly points out that the entropy patterns of different egos are different (compare, *e.g.*, panels d and e). Likewise, each ego’s patterns appear fairly persistent; however, there is more variation here, especially in the morning and at night when the call frequency is low and the entropy measures are as a result noisy. However, in general, deviations of the original entropies from the reference model’s mean are statistically significant; as the reference model’s standard deviation is fairly small, the typical deviation is > 2 std’s.

We next focus on the specific alters behind the low-entropy times of day. For this, we first count the total number of calls by each ego to each alter in each interval, and rank the alters of each ego according to this number. Alter ranks based on number of calls are known to reflect both the level of emotional closeness between ego and alter (as indexed on a standard psychological 1–10 emotional closeness scale), and the frequency face-to-face contact between ego and alter: in [19] it was shown that the number of calls significantly predicts emotional closeness, for the same data set as studied in the present manuscript.

**Fig 4:**
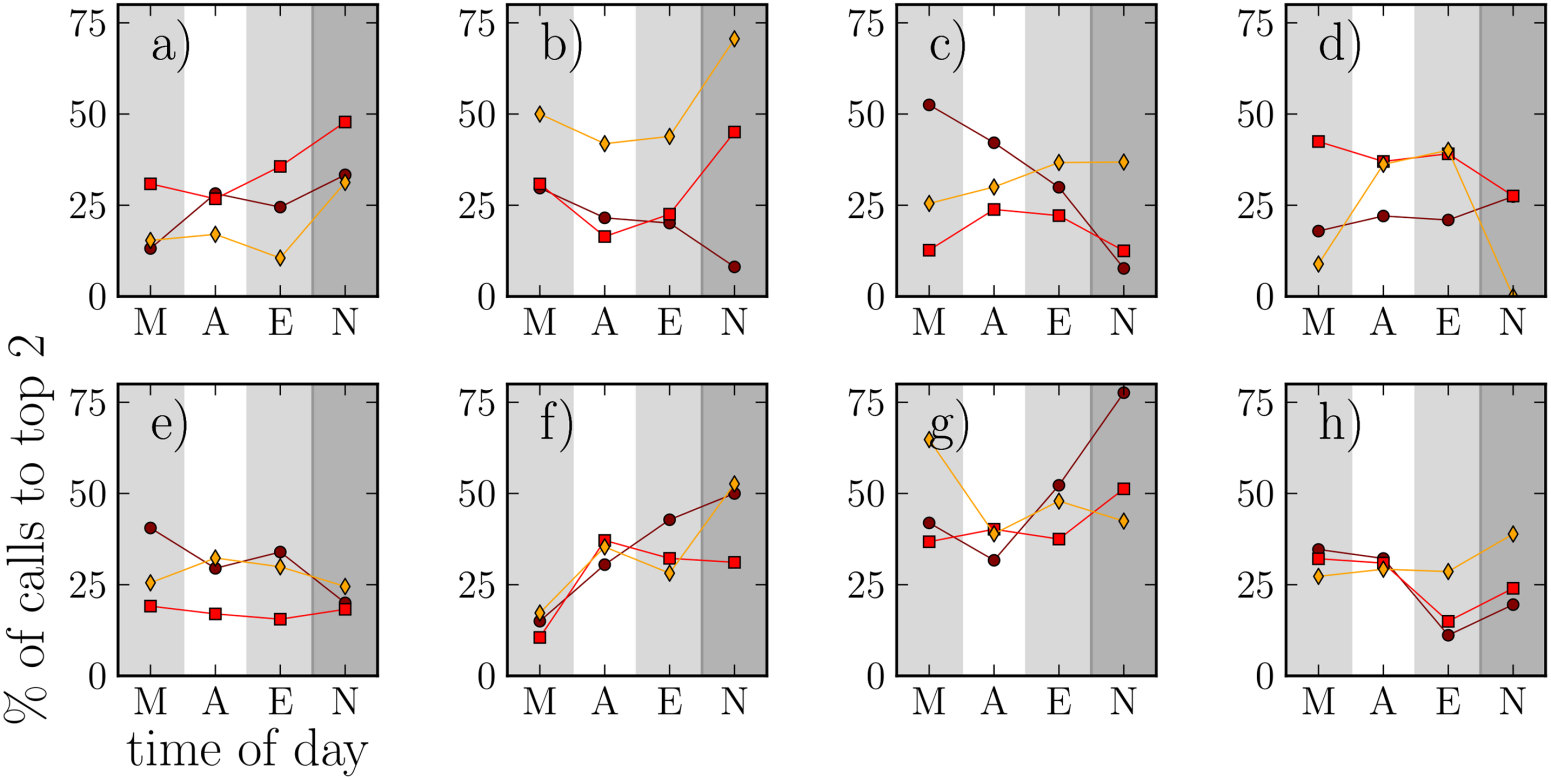
The fractions of calls to the two top-ranked alters for the same 8 individuals as in Fig 1, calculated for the same 6-hour intervals as in Fig 3 (M, A, E, N). (ο): interval *I*_1_, (◻): interval *I*_2_, (◊): interval *I*_3_.

Then, for each 6-hour interval (morning, afternoon, evening, and night) we calculate the fraction of calls directed at the two top-ranked alters (in the respective interval). These fractions are shown in Fig 4, again for the same individuals as in Fig 1. On average, it appears that the fraction of calls to two top-ranked alters of each ego increases towards late hours and is often the highest at night, when there is in general only a small number of calls and low relative entropy. The high fractions indicate that decrease of entropy towards night often comes from calls to top-ranked alters (note that we cannot rule out that this particular behaviour might be associated with the cohort’s age group and their general circumstances instead of being a general feature of human communication). Also, there is individual variation and although the top-alter fractions are often similar across intervals, in some cases, interval *I*_1_ behaves differently. This interval corresponds to the participants finishing high school and the following summer holidays, so differences in call behavior can be expected.

Because Figs 3 and 4 point towards a correlation between low entropy and calls to top-ranked alters, we next quantify this as follows: As the baseline levels and slopes of Fig 4 have a lot of variation, we take each ego and their relative entropies and fractions of calls to top-ranked alters at each 6-hour interval. Then, we compute the Pearson correlation coefficient between entropies and top-alter fractions for all egos. Out of the resulting 24 correlation coefficients, 14 were significant with *p* < 0.05, with three positive coefficients and 11 negative averaging at *r* ≈ −0.71. Thus for more than half of the egos, low entropy is clearly associated with a high fraction of calls to top alters, while for almost all the rest, no conclusive results can be drawn (note that taking a very conservative approach regarding false positives and applying the Bonferroni correction as if we were dealing with a multiple comparison test would result in 4 significant negative coefficients and 1 significant positive coefficient, with the majority of the few surviving coefficients still negative).

Since there are alter-specific communication patterns and the nature of communication depends on the time of day, we also look at call durations at different times. Here, we use data on ego and alter attributes from the conducted surveys. Previous studies have looked at gender differences in talkativeness as well as differences in usage of phones(both for landlines and mobile phones) [20–22], using data from different countries and age groups. Most of the recent studies of talkativeness suggest that men and women are similar [20, 23]. However in most studies which compare phone usage difference between men and women, women have been reported to have longer calls [24, 25]. The differences in phone usage of males and females have been linked to their different social roles [26–28]; it has also been observed that the temporal communication patterns formed by groups of male or female participants differ [29]. Here, we add two more dimensions and look at call durations at different times of the day, as well as durations of calls to different types of social links (kin or friend/acquaintance).

**Fig 5:**
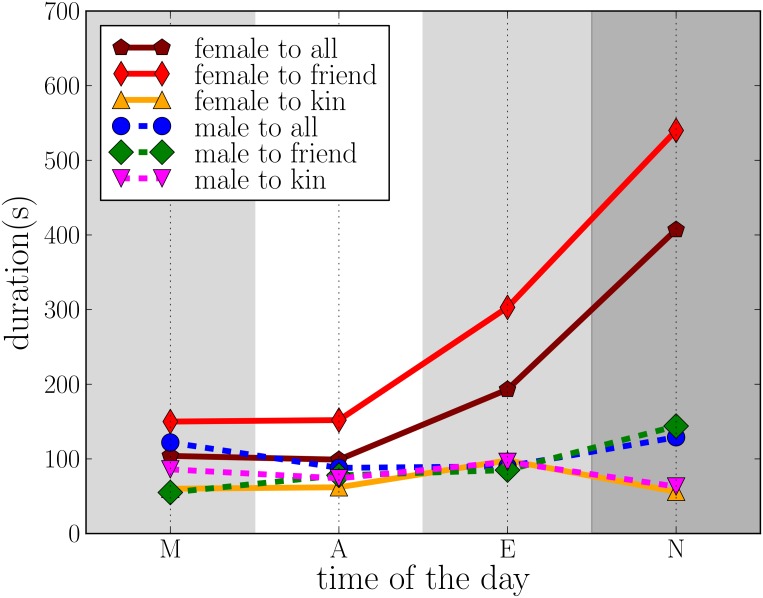
Average duration of calls made by males and females to their kin, friends, and all social contacts.

**Fig 6:**
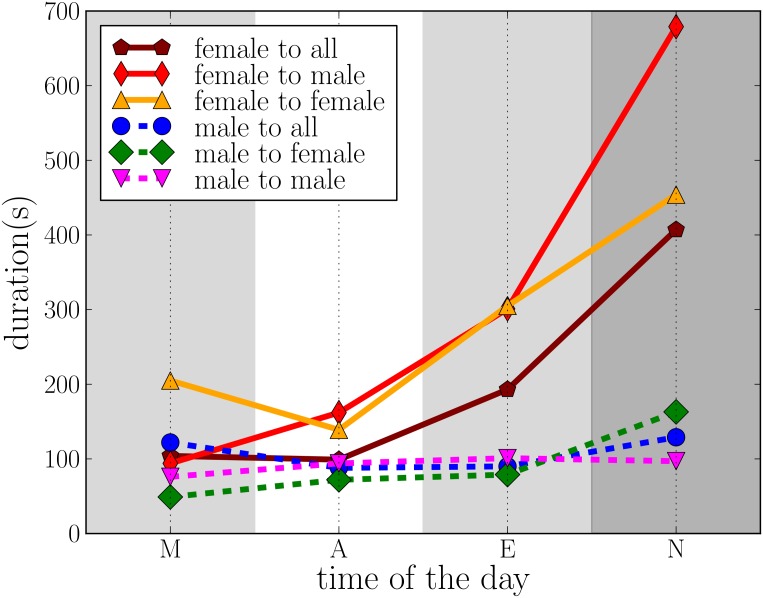
Comparison of the average duration of calls to social contacts of the same and opposite gender, separately for females and males.

Fig 5 shows that overall, the average durations of calls by females are longer than those of calls by males, and that the difference largely depends on the time of day such that it increases towards the evening and is highest at night. A closer look shows that this difference arises mostly from calls to friends. Male and female call durations to kin are fairly similar and do not depend much on the time of day. When the gender of the called alters is analysed (Fig 6), it is seen that by far the longest calls are by female egos to male alters at night; again the differences are the smallest in the afternoon, *i.e*. when all egos are typically in a similar social setting (at school, work, or university). The finding agrees with previous studies which suggest that females have different bonding strategies and use phones for different purposes compared to men [27, 30]. Since nighttime calls are often targeted at top-ranking alters (who typically are emotionally close [19]), and the egos are in their late teens and are possibly experiencing emotionally intense relationships with their romantic partners, it is likely that these long calls often relate to romantic relationships.

Since we have a relatively small sample of individuals (24 total), one might think that the high values for call durations for females in afternoons and nights might only be caused by one or few females who make very long calls in those hours. To rule out this possibility, for each individual we compare call durations made in the morning or afternoon with duration of calls made in the afternoon or night, using two-sample unequal variance t-test. We see that for 9 out of 12 females p-values for this test are less than 10^−6^, whereas only for 2 males out of 12 we have such small p-values. These results are also consistent with findings of Dong et al. [31], who have analysed a dataset of 3.9 million call records over a timespan of 2 months and have found that young females make longer calls especially in the evening.

## Discussion

In contrast to conventional studies on daily patterns and circadian rhythms in social networks that focus on aggregates of very large numbers of individuals, here we focused on a small but rich sample that combined questionnaire and mobile phone data in order to be able to explore in much greater detail features that characterise individuals’ circadian rhythms. Our focus has been on three specific issues, namely (1) whether there are individual-specific patterns of calling that mirror previously demonstrated individual patterns in the way individuals allocate their social capital to their alters; (2) whether the individual-specific patterns of calling are persistent in light of network turnover and (3) whether there are gender differences in calling patterns. We show that individuals do indeed have different daily patterns of call activity. These patterns vary beyond simple morningness/eveningness, as measured in questionnaire studies, and appear to be characteristic of the individual, in much the same way as their characteristic way of distributing their social capital among their alters [19]. Thus these individual patterns are persistent, in that the pattern of distributing calls across the day is consistent across the three time periods, despite the high degree of network turnover in the 18 months of the study, associated with leaving school and entering work or University [18, 19]. Note, however, that persistence despite social network turnover does not necessarily mean that egos’ daily patterns are entirely independent of those of their alters. Rather, it may well be that the patterns are dominated by characteristics intrinsic to egos (such as morningness/eveningness), while there is still some synchronization taking place at the same time. Our entropy results point towards this possibility: for a number of egos, closest friends are called around certain hours, and very probably, this behaviour is reciprocated (but not detectable in our data since we only have details on outgoing calls). In studies with big data—millions of anonymized call records—it is clearly seen that incoming calls trigger returned calls, which is clear evidence of synchronization [32–34]. In general, studying the synchrony of the daily patterns of connected individuals would be an interesting problem.

We also showed that there are gender differences in call duration pattern across the day: while women’s calls are generally longer than men’s calls, this was especially true during the evening and at night. Evening calls to males and to friends by female egos were especially long, and often involved calls to specific individuals, usually the top-ranked alters, who may be boyfriends.

Given that humans naturally spend the night asleep, the tendency for calls to exhibit a striking diurnal periodicity is not, of itself, especially surprising, of course. However, in our sample, the vast majority of calls were made between midday and late evening, with the bulk of these occurring in the 6-hour slot between 12AM-6PM Fig 1. It is notable that, despite our essentially diurnal nature, rather few calls were made before midday. Therefore, it is possible that most calls made by our cohort of subjects are social rather than functional (i.e. work or leisure-activity related). Interestingly, for many egos, communication at late evening and night frequently involves their closest friends. One could ask whether our social behaviour is in general different at night and during daylight hours. Wiessner [35] reported that certain types of conversations (notably story-telling and social conversations) are much more common during the evening than during the day among !Kung San hunter gatherers, with conversations involving economic matters or social criticism taking place mainly during daylight hours. In her sample, 81% of evening fireside conversations involved storytelling (relaying of adventures or experiences, especially in far off places, or tales about myths, social conventions and rituals, experiences during trance states or real life travels).

Within this broad pattern, the individual differences in the distribution of calling, and particularly the persistence of these individual differences in the light of social network turnover, are strongly suggestive of some kind of personality characteristic. It is possible that these differences in personal style simply reflect individual differences in circadian pattern [4–6], and are a consequence of the fact that some individuals are more active in the morning and others more active in the evening. It is perhaps less likely that the calling patterns are due to differences in individual sleep/wake cycles, since the demands of the working day are likely to have required everyone to be active in the morning and even early risers are unlikely to have gone to bed by 6PM (note that circumstances such as unemployment do have effects on daily cycles of individuals, see [17]). However, it could be that, physiologically, morning people are more likely to feel motivated to be socially engaged in the daytime and evening people more likely to be so in the evening. The fact that some individuals find the evening hours particularly attractive, while others prefer the day, remains intriguing in this context and obviously merits more detailed investigation.

Notwithstanding the fact that some individuals are night-oriented and others day-oriented, it seems that many (though not all) egos prefer to call certain alters at night. These are typically the one or two individuals (mainly males and friends) that have special status for the ego (Fig 4). We know from our detailed questionnaire data that the individuals that egos call most often are those to whom they are emotionally closest, and those they have the most frequent face-to-face contact with [19]. It seems that this is especially characteristic of female egos, and much less so of male egos. Unlike women, men do not call either their girlfriends or their same-sex best friends for long chats in the evenings (even though their girlfriends may call them). This striking sex difference in who actively makes the effort to call is reminiscent of the finding reported by Palchykov et al. [36], for a very large cellphone dataset, that younger women (in particular) are much more proactive in calling their primary male contact than are men. This striking difference between the two sexes may reflect women’s more intensely social nature compared to men.

For both sexes, it was much less common to call kin during the evening. This would reinforce the claim that relationships with kin are less fragile than those with friends, and hence require less persistent and less special servicing [18]. Reserving calls to these individuals for times of the day when they are, or might seem to be, more intimate may reinforce the sense that the relationship is special. In effect, kin relationships come for free by virtue of the fact that they are kin and ego is embedded in a densely interconnected web of relationships with them, and therefore require less active maintenance. In contrast, the quality of friendships deteriorates rapidly (within months) in the absence of sufficiently frequent contact [18, 37, 38].

A strength of our study was that it combined detailed mobile phone records with questionnaire data. Thus we have information on the nature of the relationship between egos and the alters they are calling, in terms of gender, kinship and emotional intensity. Further, we know from previous findings that the number and duration of phone calls relates to the emotional intensity of the relationship, as well as the level of social activity [18, 19]. This dataset therefore allows for analysis of the social nature of circadian rhythms, rather than simply examining aggregate analyses of mobile phone activity or broad scale questionnaire data. Even though the sample size is relatively small due to the intensive, longitudinal data collection, the nature of the data allows us to add a level of individual detail on identity of the callers and callees. Our results may help interpreting the results of big data studies (e.g. on related themes of information propagation and temporal communication motifs [29, 32, 33, 39].

## Materials and Methods

### Our data and its use

Our dataset includes 18 months of outgoing call and text records of the 24 individuals. In this study we have only used the call records (both to mobile phones and landlines). In addition to this mobile phone data, participants completed 3 questionnaires about the people in their social network at the beginning (month 0), in the middle (month 9) and at the end (month 18) of the study. They identified each contact (alter) as kin or non-kin, and provided all the different phone numbers that one contact might possibly have. Therefore these records are very comprehensive and do not miss calls or texts because an alter has several phone numbers (with multiple phone providers) and/ or uses a landline. In the social network questionnaires, participants provided information about each alter, including gender, how emotionally close they are to the person and the frequency of face-to-face contact with the person. The data on phone calls was obtained from the fully time-stamped, itemised monthly bills. These itemised bills were accessed by one of the researchers (S.G.B.R.) from the individual online accounts the participants had with the mobile telephone operator, with the written consent of the participants. As compensation for taking part in the study, the participants were given a mobile phone with an 18 month contract from a major UK mobile telephone operator. The line rental for the mobile phone was paid for and included 500 monthly free voice minutes (to landlines or mobiles) and unlimited text messages. The participants, questionnaire and mobile phone datasets are described fully in previous publications [18, 19]. Anonymized, aggregated data is available online and the detailed time-stamped data used in this study is available on request. This study was approved by the Ethics Review Committee of the University of Liverpool (the institution at which the data collection phase of this project was originally initiated). Written informed consent was obtained from all participants prior to data collection.

### Calculating daily patterns

To calculate daily patterns of each ego, we have taken data from all days in the time interval of interest and have allocated each call to a 6-hour time bin based on its time stamp. We then count total number of events of each hour and divide it by total number of events (of that ego) during the the time interval, to get the fraction of calls in that hour. In each time interval, we only used data of complete weeks in that time interval.

### Measuring similarity of patterns

The Jensen-Shannon divergence is a measure of the difference of two probability distributions. It is a form of Kullback-Leibler divergence (KLD); unlike KLD, it works for probability distributions that contain zero-valued elements. The JSD for two discrete probability distributions *P*_1_ and *P*_2_ follows the formula $JSD\;\left(P_{1},P_{2}\right)=H\;\left(\frac{1}{2}P_{1}+\frac{1}{2}P_{2}\right)-\frac{1}{2}\left[H\;\left(P_{1}\right)-H\;\left(P_{2}\right)\right]$, where *P*_*i*_ = *p*_*i*_(*t*) and *p*_*i*_(*t*) is the fraction of calls at each (binned) time of the day, and *H* is the Shannon entropy (*H*(*P*) = −∑*p*(*t*)log*p*(*t*)).

We have also used the *l* ^2^-norm as a way to verify our results calculated using JSD. ℓ^2^-norm is a similarity measure of two distributions, which is defined as: $\ell^{2}=\sqrt{\sum |p_{1}\left(t\right)-p_{2}{\left(t\right)|}^{2}}$.

### Entropy patterns and relative entropies

We calculate the call entropy for a given hour (or range of hours) as follows: first, the fraction of calls out of all calls to each alter *a*, *p*_*a*_, is counted for the specified hour (range of hours). Then, the call entropy for this hour (range of hours) is computed as *H*_orig_ = −∑_*a*_ *p*_*a*_ log*p*_*a*_. In order to obtain the relative entropy, we repeatedly shuffle the original data as follows: for each week, the times and recipient alters of all calls are randomly shuffled. This reference model corresponds to a situation where the original call frequency pattern and the number of calls to each alter are the same, but no preference is shown to any specific alter at any specific time. Then, for each shuffled set of data, we calculate call entropy similarly as for the original data, and average over *N* = 1,000 realizations to get the average reference entropy 〈*H*_ref_〉. Finally, the relative entropy is obtained as *H*_rel_ = *H*_orig_/〈*H*_ref_〉. The shuffling for the reference model is done on a weekly basis in order to minimize the effects of long-term dynamics, such as declining numbers of calls to alters, or alters appearing for the first time within the studied 6-month interval.
